# Damage control for subclavian artery injury

**DOI:** 10.1590/1677-5449.200007

**Published:** 2020-09-14

**Authors:** Adenauer Marinho de Oliveira Góes, Mariana Pereira Maurity, Carlos Alberto Costa do Amaral

**Affiliations:** 1 Cirurgia Vascular, Hospital Metropolitano de Urgência e Emergência – HMUE, Ananindeua, PA, Brasil.; 2 Faculdade de Medicina, Universidade Federal do Pará – UFPA, Belém, PA, Brasil.; 3 Cirurgia Geral, Hospital Metropolitano de Urgência e Emergência – HMUE, Ananindeua, PA, Brasil.; 4 Curso de Medicina, Universidade Faculdade Metropolitana da Amazônia – UNIFAMAZ, Belém, PA, Brasil.

**Keywords:** chest traumas, subclavian artery, subclavian steal syndrome, vascular system injuries

## Abstract

Mortality from penetrating traumas involving the subclavian vessels can be as high as 60% in pre-hospital settings. Operating room mortality is in the range of 5-30%. This paper presents a case in which a strategy for damage control was employed for a patient with an injury to the origin of the left subclavian artery, using subclavian ligation, with no need for any other intervention, and maintaining viability of the left upper limb via collateral circulation. The authors also review surgical approaches and treatment strategies with a focus on damage control in subclavian vessel injuries.

## INTRODUCTION

Mortality from penetrating traumas involving subclavian vessels can reach 60% in prehospital settings, making speedy transportation to a trauma center a decisive factor. Intraoperative mortality is in the range of 5-30%.^1^^-^^4^

The proximity of neurovascular structures, hematoma, anatomic abnormalities and the need for adequate exposure make surgery to treat such traumas challenging.^3^^-^^5^ Postoperative mortality varies from 5 to 40%,^2^^,^^3^ and postoperative complications affect 24%.^4^

The correct surgical approach is of paramount importance. An incision over the clavicle, extended to the deltopectoral groove, may be sufficient for more distal injuries, but injuries to the intrathoracic segment demand a thoracotomy.^1^

Right-side injuries can be approached via a median sternotomy, with anterior cervical and/or right supraclavicular extension if necessary. Left-side injuries are better explored via an anterolateral thoracotomy between the third and fifth intercostal spaces, which can be expanded with a median sternotomy and, if necessary, extended with a supraclavicular incision (open book/trapdoor access).^1^^,^^3^^,^^5^

Arterial injuries can be corrected with sutures, end-to-end anastomosis or grafting (autologous or prosthetic).^1^^,^^5^^,^^6^ In serious cases, a temporary intravascular shunt can be used for damage control until stabilization is achieved^6^^,^^7^ and, in extreme cases, the subclavian artery can be ligated with little risk of ischemia.^6^^,^^8^^,^^9^

This article describes a damage control strategy employed in a patient with an injury to the origin of the left subclavian artery.

### Part I – Clinical situation

The case described is of a 24-year-old male patient, victim of a gunshot wound to the left infraclavicular region at the parasternal line. He arrived at the hospital with patent airways, an asymmetrical thorax, thoracic wound with air leak, absent vesicular murmur in the left hemithorax, heart rate (HR) of 135 bpm, sweating profusely, with cold skin, and blood pressure (BP) of 80 x 50 mm Hg. Pleural drainage revealed a large volume hemothorax (1,400 mL).

After initial treatment, BP of 110 x 80 mm Hg and HR of 122 bpm were achieved, but the patient developed respiratory distress and his level of consciousness reduced. He was subjected to oral endotracheal intubation and transferred to the operating room for thoracotomy.

### Part II – What was done

A left anterolateral thoracotomy was performed at the fifth intercostal space. After removal of a large clot from the pleural cavity, a through-and-through wound was identified in the superior pulmonary lobe (without active bleeding) and a large hematoma involving the upper mediastinum, which could not be explored because of the operating field obtained. The decision was taken to expand the access with a transverse sternotomy and extension to a right anterolateral thoracotomy from the fifth intercostal space to the parasternal line.

Proximal control of the supra-aortic trunks was obtained by performing a longitudinal pericardiotomy, before manipulating the hematoma. During this phase of surgery, intense bleeding from the injury started spontaneously. This was partially controlled by “en masse” application of a Satinsky clamp to the structures adjacent to the source of bleeding.

At this point, the patient suffered cardiac arrest with ventricular fibrillation, and resuscitation maneuvers were initiated, including descending aorta cross-clamping, internal cardiac compressions and defibrillation and intravenous adrenaline. Sinusoidal rhythm was reestablished after 4 minutes.

Dissection was resumed and a partial avulsion of the left subclavian artery close to its origin was identified. In view of the severity of the patient’s condition, the decision was taken to ligate the artery. Hemostasis was reviewed and bilateral thoracic drains were installed, followed by closure. The patient was transferred to the intensive care unit (ICU) in a critical state, on vasoactive drugs. He spent 20 days in the ICU and was discharged from hospital on the 35th day after the operation.

**Figure 1:**
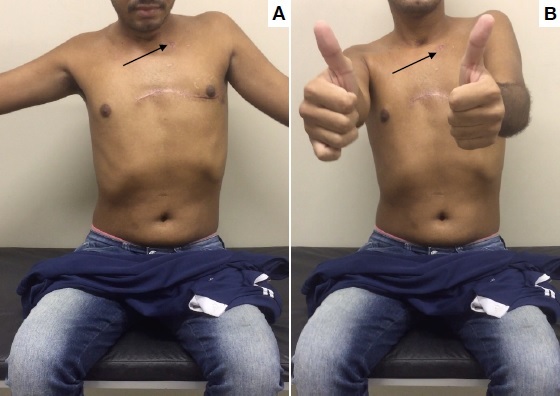
Photographs taken 3 months after surgery, showing functional recovery of the left upper limb. The arrow indicates the site of the gunshot wound.

**Figure 2:**
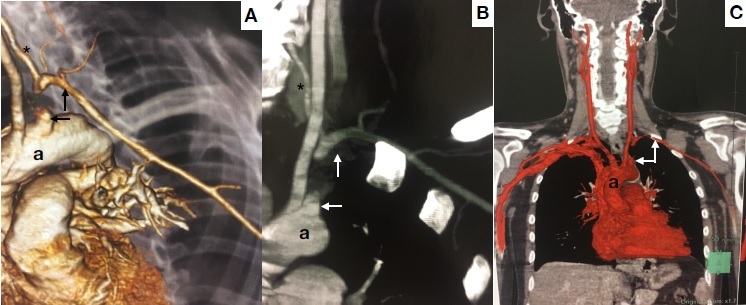
Angiotomography conducted 3 months after surgery. The reconstructions in 1.A, 1.B, and 1.C, show: A: aorta; *left vertebral artery; arrows indicate the left subclavian artery ligated at the origin and the point at which it becomes opaque again.

The patient has been in outpatients follow-up for 10 months; is free from neurological sequelae associated with the cardiac arrest, and left arm function has recovered well, with systolic blood pressure at 80% of the value measured in the contralateral limb and a palpable radial pulse, although with reduced amplitude (Figure 1). Angiotomography conducted 3 months after surgery showed that vascularization of the left upper limb is via retrograde filling of the left vertebral artery (Figure 2).

## DISCUSSION

Vascular injuries in the cervico-thoracic outlet are rare and up 90% of cases are caused by penetrating injuries; 2 to 4% of these have subclavian vessels involvement.^1^^,^^4^^,^^10^

In stable patients, surgical access can be planned taking into consideration the results of imaging exams such as angiotomography and, occasionally, angiography and Doppler ultrasonography. However, patients whose physiological parameters are in decline should undergo surgery immediately, choosing the approach on the basis of the mechanism of trauma.^1^^-^^3^^,^^11^

Proximal injuries to the left subclavian artery are classically approached via an anterolateral thoracotomy incision through the third intercostal space, but in this case the wound was within the “cardiac box” and so the possibility of heart injuries had to be considered.^12^

**Figure 3:**
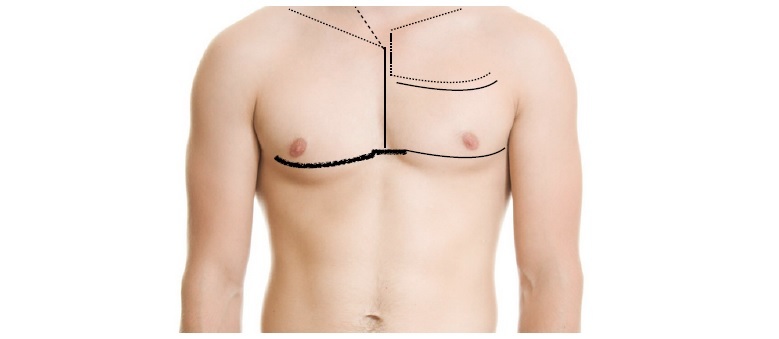
Approaches for treatment of thoracic vascular injuries. Longitudinal continuous line: median sternotomy, the broken and dotted straight lines indicate possible cervical or right supraclavicular extensions. Solid curved lines indicate left anterolateral thoracotomy incisions over the fifth or third intercostal spaces; the dotted lines on the left hemithorax illustrate extension to a longitudinal sternotomy and supraclavicular incision (open book); while the thicker line indicates extension to a bilateral thoracotomy with transverse sternotomy.

Anterolateral thoracotomy through the fifth space is the classic approach for penetrating chest injuries in unstable patients and extension across to the contralateral thoracic cavity provides access to both pleural cavities and the mediastinum.^13^^-^^15^ Surgical approaches for traumatic vascular injuries are illustrated in Figure 3.

In stable patients, in some cases with a preoperative radiological diagnosis and endovascular resources available, an angioplasty balloon can be inflated in the subclavian artery to achieve proximal vascular control.^16^^,^^17^ However, in the case described, with near-total avulsion of the left subclavian artery at its origin, any attempt to cross the lesion with a guidewire would have been very unlikely to succeed and there would probably not have been enough space to inflate the balloon proximally to the injury. During surgical exploration, the safest technique for avoiding hemorrhage is to obtain proximal vascular control before dealing with the injury, which was exactly the objective of dissection of the intrapericardial segments of the supra-aortic trunks. Unfortunately, bleeding started while the attempt to achieve proximal control was still underway.

A damage control strategy comprises operating tactics that shorten the duration of surgery, remaining within physiological limits and increasing the patient’s chances of survival.^18^^,^^19^ The strategy is generally conducted in three stages: 1) surgical control of life-threatening injuries, achieving hemostasis and preventing contamination of cavities; 2) resuscitation and ICU care, and 3) definitive surgical treatment.^18^^,^^19^ In the case described, revascularization was unnecessary because perfusion of the limb was adequate.

Another damage control strategy is to use a vascular shunt, which can be made using a segment of tubular material with a compatible diameter, such as a nasogastric tube,^7^^,^^20^ providing temporary perfusion of the limb/organ. However, the very small neck at the origin from the aortic arch would have made insertion extremely difficult. In view of the imminent risk of death, ligature of the subclavian artery was chosen, since, when proximal to the origin of the vertebral artery, this rarely provokes decompensated ischemia of the limb.^6^^,^^8^^,^^9^

A review comparing management of penetrating arterial injuries caused by cervical-thoracic traumas in two distinct periods in the same hospital (modern, 2000-2013, and previous, 1974-1988) concluded that endovascular treatment was only used in the modern period, in stable patients with pseudoaneurysms. Ligature was rarely employed in either period, only used when there was a risk of death, and shunts were only used in the modern period, as an alternative to vessel ligation, when possible.^21^

In a different patient sample, of twenty cases of subclavian artery injury, an association with brachial plexus injury was observed in 55% of cases. Additionally, in that study just one patient underwent arterial ligature, and the outcome was death.^22^

Another study, with 38 cases of subclavian/axillary arteries injuries, analyzed types of treatment and outcomes: in 5 cases, ligature of the artery was performed because of the clinical conditions at the time of surgery. There were two deaths, but no signs of ischemia of the limb in the three survivors.^23^

Ligature is reserved for critical cases, when cardiac arrest secondary to hypovolemia may occur in the operating room. If this does occur, thoracotomy resuscitation maneuvers are required, such as clamping the descending aorta, longitudinal pericardiotomy, to enable internal cardiac compressions, at 60-100 bpm, and direct defibrillation, which should be performed with a charge of 10-30 Joules.^8^^,^^14^^,^^24^^,^^25^ All of these maneuvers were performed in the case described here.

In this case, the absence of limb ischemia is because of subclavian steal syndrome, originally described when there is stenosis in the proximal artery. The syndrome demonstrates a protective effect between the subclavian artery and the vertebrobasilar system.^9^^,^^11^

In this situation, retrograde blood flow though the vertebral artery occurs if the blood pressure distally to the injury reaches a lower level than the pressure at the basilar artery, via the contralateral vertebral artery and the circle of Willis. This reverse flow through the vertebral artery may be intermittent or continuous, depending on the degree of stenosis, supplying hypoperfused vascular beds.^9^^,^^11^ Although revascularization of the patient’s arm could be scheduled, the trend is to keep the patient in follow-up and maintain conservative management, since he remains asymptomatic.

Endovascular treatment eliminates risks inherent to surgical dissection. Although the literature reports success rates of up to 95% of cases, the majority are pseudoaneurysm and arteriovenous fistula cases and not acute phase treatments; in such cases, it is recommended that the stent should be deployed without covering the origin of the vertebral artery, thereby reducing the risk of stroke.^16^^,^^17^ The hemodynamic instability and the anatomic characteristics of the injury already described mean that in such situations, endovascular techniques are not the first option. Moreover, endovascular resources were not available.

Surgeons should be familiar with damage control strategies, which are not only useful in trauma cases, but also when faced with complications during elective surgery. In this context, ligature of the subclavian artery, proximal to the origin of the vertebral artery, is a viable option involving a relatively low risk of insufficient upper limb perfusion.
