# Supplementary material for: Damage control for subclavian artery injury
Source: J Vasc Bras. 2020 Sep 14;19:e20200007. doi: 10.1590/1677-5449.200007 (PMC8276654; doi:10.1590/1677-5449.200007)
Supplement: Translation

# Translation: Controle de danos para lesão de artéria subclávia

**Authors:** Adenauer Marinho de Oliveira Góes, Mariana Pereira Maurity, Carlos Alberto Costa do Amaral

**Affiliations:** 1 Cirurgia Vascular, Hospital Metropolitano de Urgência e Emergência – HMUE, Ananindeua, PA, Brasil.; 2 Faculdade de Medicina, Universidade Federal do Pará – UFPA, Belém, PA, Brasil.; 3 Cirurgia Geral, Hospital Metropolitano de Urgência e Emergência – HMUE, Ananindeua, PA, Brasil.; 4 Curso de Medicina, Universidade Faculdade Metropolitana da Amazônia – UNIFAMAZ, Belém, PA, Brasil.

**Keywords:** traumatismos torácicos, artéria subclávia, síndrome do roubo subclávio, lesão do sistema vascular

## Abstract

Os traumas penetrantes de vasos subclávios atingem mortalidade de até 60% em um cenário pré-hospitalar. A mortalidade no intraoperatório varia de 5-30%. Apresenta-se um caso de estratégia de controle de danos para um paciente com lesão na origem da artéria subclávia esquerda, através de ligadura, sem necessidade de outra intervenção, mantendo a viabilidade do membro superior esquerdo por meio de circulação colateral. Os autores fazem uma revisão sobre vias de acesso e estratégias de tratamento com ênfase em controle de danos para lesões de vasos subclávios.

## INTRODUÇÃO

Traumas penetrantes de vasos subclávios atingem mortalidade de até 60% no cenário pré-hospitalar, tornando a rapidez de acesso ao centro de trauma um fator decisivo. A mortalidade intraoperatória varia de 5-30%^1^^-^^4^.

A proximidade de estruturas neurovasculares, hematoma, alterações anatômicas e a necessidade de exposição adequada tornam desafiadora a cirurgia para tratamento desses traumas^3^^-^^5^. A mortalidade pós-operatória varia entre 5 e 40%^2^^,^^3^, e complicações pós-operatórias chegam a 24%^4^.

O acesso cirúrgico adequado é fundamental. Uma incisão sobre a clavícula, com extensão para o sulco deltopeitoral, pode ser suficiente para lesões mais distais; no entanto, lesões do segmento intratorácico requerem toracotomia^1^.

Lesões à direita são abordadas por esternotomia mediana, se necessário com extensão cervical anterior e/ou supraclavicular direita. Lesões à esquerda são mais bem exploradas via toracotomia anterolateral entre o 3º e o 5º espaços intercostais, podendo ser ampliada com esternotomia mediana e, se necessário, estendida com incisão supraclavicular (acesso em livro aberto/alçapão)^1^^,^^3^^,^^5^.

As lesões arteriais podem ser corrigidas com sutura, anastomose término-terminal ou enxerto (autólogo ou com prótese)^1^^,^^5^^,^^6^. Em casos graves, pode ser implantado um shunt intravascular temporário como controle de danos até que a estabilização seja atingida^6^^,^^7^ e, em casos extremos, a artéria subclávia pode ser ligada com baixo risco de isquemia^6^^,^^8^^,^^9^.

Neste artigo, é descrita uma estratégia de controle de danos em um paciente com lesão na origem da artéria subclávia esquerda.

### Parte I - Situação clínica

O caso descrito é de um paciente masculino, 24 anos, vítima de ferimento por arma de fogo em região infraclavicular esquerda na linha paraesternal. Chegou ao hospital com vias aéreas pérvias, tórax assimétrico, ferimento torácico com escape aéreo, murmúrio vesicular abolido em hemitórax esquerdo, frequência cardíaca (FC) 135 bpm, sudoreico, pele fria, pressão arterial (PA) 80 x 50 mm Hg. A drenagem pleural detectou hemotórax volumoso (1.400 mL).

Após o atendimento inicial, obteve-se PA: 110 x 80 mm Hg e FC 122 bpm; a seguir, houve desconforto respiratório e rebaixamento do nível de consciência. O paciente foi submetido a intubação orotraqueal e encaminhado ao bloco cirúrgico para toracotomia.

### Parte II – O que foi feito

O paciente foi submetido a toracotomia anterolateral esquerda no 5º espaço intercostal. Após a remoção de coágulo volumoso da cavidade pleural, identificou-se ferimento transfixante no lobo pulmonar superior (sem sangramento ativo) e grande hematoma em mediastino superior, cuja exploração não foi possível pelo campo operatório obtido. Optou-se por ampliar o acesso com esternotomia transversa e prolongamento para toracotomia anterolateral direita no 5º espaço intercostal até a linha paraesternal.

Para obter controle proximal dos troncos supra-aórticos, antes da manipulação do hematoma, realizou-se pericardiotomia longitudinal. Durante esse tempo cirúrgico, houve destamponamento espontâneo da lesão com sangramento intenso, sendo parcialmente controlado com *clamp* de Satinsky englobando em massa as estruturas adjacentes ao foco hemorrágico.

Nesse momento, houve parada cardíaca com fibrilação ventricular, sendo procedidas manobras de reanimação que incluíram clampeamento da aorta descendente, massagem e desfibrilação cardíacas internas e adrenalina endovenosa. Recuperou-se ritmo sinusal após 4 minutos.

A dissecção prosseguiu, identificando-se avulsão parcial da artéria subclávia esquerda justa à sua origem. Devido à gravidade do quadro, optou-se pela ligadura da artéria. Realizou-se revisão da hemostasia, drenagem torácica bilateral e síntese. O paciente foi encaminhado à unidade de terapia intensiva (UTI) em estado grave e usando drogas vasoativas. Permaneceu na UTI por 20 dias e recebeu alta hospitalar no 35º dia de pós-operatório.

O paciente encontra-se em acompanhamento ambulatorial há 10 meses; não apresentou sequelas neurológicas associadas à parada cardíaca e houve boa recuperação funcional do membro superior esquerdo, com pressão arterial sistólica de 80% em relação àquela aferida no membro contralateral e pulso radial palpável, embora de amplitude diminuída (Figura 1). A vascularização do membro superior esquerdo, conforme documentado por angiotomografia realizada no 3º mês do pós-operatório, ocorre por enchimento retrógrado da artéria vertebral esquerda (Figura 2).

**Figura 1:** Fotografias realizadas no 3º mês do pós-operatório, demonstrando a recuperação funcional do membro superior esquerdo. A seta aponta o ferimento por projétil de arma de fogo.

**Figura 2:** Angiotomografia realizadas no 3º mês pós-operatório. Nas reconstruções em 1.A, 1.B e 1.C, é possível observar: A: aorta; *artéria vertebral esquerda; as setas apontam a artéria subclávia esquerda ligada na origem e sua reopacificação.

## DISCUSSÃO

Lesões vasculares cérvico-torácicas são raras e, em até 90% dos casos, causadas por ferimentos penetrantes, com acometimento de vasos subclávios em 2 a 4% desses traumatismos^1^^,^^4^^,^^10^.

Em pacientes estáveis, o acesso cirúrgico pode considerar resultados de exames de imagem como angiotomografia e, ocasionalmente, angiografia e eco-Doppler. Pacientes com deterioração dos parâmetros fisiológicos devem ser submetidos a cirurgia imediata, com acesso baseado no mecanismo de trauma^1^^-^^3^^,^^11^.

Lesões proximais da artéria subclávia esquerda são classicamente abordadas por toracotomia anterolateral no 3º espaço intercostal, porém, neste caso, o ferimento está contido na “zona de Ziedler” e a possibilidade de lesões cardíacas deve ser considerada^12^.

A toracotomia anterolateral no 5º espaço é a abordagem clássica para ferimentos torácicos penetrantes em pacientes instáveis e a ampliação para a cavidade torácica contralateral permite abordar as duas cavidades pleurais e o mediastino^13^^-^^15^. Vias de acesso para lesões vasculares traumáticas são ilustradas na Figura 3.

**Figura 3:** Vias de acesso para lesões vasculares torácicas. Linha contínua longitudinal: esternotomia mediana, as linhas tracejada e pontilhada indicam possíveis prolongamentos cervicais ou supraclavicular direita. Linhas arqueadas sólidas indicam toracotomias anterolaterais no 5º ou 3º espaço intercostal esquerdo; a linhas pontilhadas no hemitórax esquerdo demonstram prolongamento para esternotomia longitudinal e incisão supraclavicular (livro aberto); o prolongamento indicado pela linha mais grossa indica a bitoracotomia com esternotomia transversa.

Em pacientes estáveis, havendo diagnóstico radiológico da lesão no pré-operatório e recursos endovasculares disponíveis, em alguns casos, um balão de angioplastia pode ser insuflado na artéria subclávia para o controle vascular proximal^16^^,^^17^. Entretanto, neste caso, devido à avulsão quase completa da subclávia esquerda rente à sua origem, dificilmente se conseguiria ultrapassar a lesão com um fio-guia e, provavelmente, não haveria espaço suficiente para insuflar o balão proximalmente à lesão. Durante uma exploração cirúrgica, a medida mais segura para evitar o destamponamento da lesão é obter controle vascular proximal antes de abordá-la; justamente o que se buscou ao dissecar as porções intrapericárdicas dos troncos supra-aórticos. Infelizmente, o sangramento ocorreu enquanto se tentava obter este controle proximal.

A estratégia de controle de danos consiste em táticas operatórias que abreviem a cirurgia, conservando os limites fisiológicos e aumentando a chance de sobrevida do paciente^18^^,^^19^. A estratégia habitualmente se desenvolve em três etapas: 1ª) controle cirúrgico de lesões ameaçadoras da vida, através de hemostasia e interrupção da contaminação de cavidades; 2ª) reanimação e cuidados em UTI e 3ª) tratamento cirúrgico definitivo^18^^,^^19^. No caso apresentado, como a perfusão do membro se manteve compensada, a revascularização não foi necessária.

Outra estratégia de controle danos é o uso de um shunt vascular, que pode ser realizado com um segmento de material tubular de diâmetro compatível, como sonda nasogástrica^7^^,^^20^, promovendo perfusão temporária do membro/órgão. No entanto, o exíguo colo em relação ao arco aórtico acarretaria grande dificuldade para sua inserção. Diante do risco iminente de óbito, optou-se pela ligadura da artéria subclávia, a qual, quando proximal à origem da artéria vertebral, raramente provoca isquemia descompensada do membro^6^^,^^8^^,^^9^.

Uma pesquisa comparando o manejo de lesões arteriais penetrantes no trauma cérvico-torácico entre duas épocas (moderna, 2000-2013, e prévia, 1974-1988) no mesmo hospital concluiu que o tratamento endovascular ocorreu somente na era moderna, em pacientes estáveis com pseudoaneurismas. A ligadura foi raramente utilizada nos dois períodos, apenas quando havia risco de óbito, e a confecção de shunt temporário ocorreu somente na época moderna como alternativa à ligadura do vaso, quando possível^21^.

Em outra casuística, de vinte casos de lesão de artéria subclávia, demonstrou-se associação com lesão de plexo braquial em 55% dos casos. Além disso, nesse trabalho, apenas um paciente foi submetido a ligadura arterial, porém evoluiu a óbito^22^.

Outro estudo, com 38 casos de traumas de artérias subclávia/axilar, analisou tipos de tratamento e desfechos: em 5 casos, realizou-se ligadura da artéria devido a condições clínicas no momento da cirurgia. Houve duas mortes, porém não houve sinais de isquemia do membro nos três sobreviventes^23^.

A ligadura fica reservada para casos graves, quando parada cardíaca por hipovolemia pode ocorrer no intraoperatório. Caso isso ocorra, estão indicadas manobras de toracotomia de reanimação, como clampeamento da aorta descendente, pericardiotomia longitudinal, permitindo massagem cardíaca interna, com 60-100 bpm, e desfibrilação direta, que deve ser feita com a carga de 10-30 Joules^8^^,^^14^^,^^24^^,^^25^. Todas essas manobras foram executadas no caso descrito.

Neste caso, a ausência de isquemia do membro pode ser explicada pela síndrome do roubo da subclávia, originalmente descrita quando há estenoses no trecho proximal da artéria. Essa síndrome demonstra efeito protetor entre a artéria subclávia e o sistema vertebro-basilar^9^^,^^11^.

Neste contexto, há fluxo retrogrado através da artéria vertebral caso a pressão arterial distalmente à lesão atinja níveis inferiores aos transmitidos à artéria basilar pela vertebral contralateral e pelo polígono de Willis. O fluxo inverso pela artéria vertebral pode ser intermitente ou contínuo, dependendo do grau da estenose, suprindo leitos hipoperfundidos^9^^,^^11^. Apesar de uma revascularização do membro do paciente em questão poder ser programada, como o paciente segue assintomático, a tendência é manter acompanhamento com conduta conservadora.

O tratamento endovascular elimina riscos inerentes à dissecção cirúrgica. Embora a literatura refira sucesso de até 95% dos casos tratados, a maioria consiste em tratamentos fora da fase aguda, como pseudoaneurismas e fístulas arteriovenosas; nesses casos, recomenda-se evitar que o stent implantado cubra a origem da artéria vertebral, diminuindo os riscos de acidente vascular encefálico^16^^,^^17^. Tanto a instabilidade hemodinâmica quanto as características anatômicas anteriormente descritas para a lesão fazem com que, para situações como a deste caso, a técnica endovascular não seja a primeira opção; além disso, recursos endovasculares não estavam disponíveis.

O cirurgião deve conhecer estratégias de controle de danos, úteis não apenas no trauma, mas diante de complicações de cirurgias eletivas. Nesse contexto, a ligadura da artéria subclávia, proximalmente à origem da artéria vertebral, é uma alternativa viável que acarreta risco relativamente baixo de descompensação da perfusão do membro superior.
